# Spatial ICA reveals functional activity hidden from traditional fMRI GLM-based analyses

**DOI:** 10.3389/fnins.2013.00154

**Published:** 2013-08-27

**Authors:** Jiansong Xu, Marc N. Potenza, Vince D. Calhoun

**Affiliations:** ^1^Department of Psychiatry, Yale School of Medicine, Yale UniversityNew Haven, CT, USA; ^2^Child Study Center, Yale School of Medicine, Yale UniversityNew Haven, CT, USA; ^3^Department of Neurobiology, Yale School of Medicine, Yale UniversityNew Haven, CT, USA; ^4^The Mind Research NetworkAlbuquerque, NM, USA; ^5^Department of Electrical and Computer Engineering, The University of New MexicoAlbuquerque, NM, USA

**Keywords:** independent component analysis (ICA), general linear model (GLM), functional magnetic resonance imaging (fMRI), functional network of the brain, functional connectivity

Independent component analysis (ICA) is a signal processing technique using higher-order statistics to extract signals by unmixing signal mixtures. McKeown et al. (1998) introduced spatial ICA (sICA) into functional magnetic resonance imaging (fMRI) study in the late 1990s. SICA assumes that fMRI signal from each voxel represents a linear mixture of source signals, separates this signal mixture into spatially independent source signals using higher-order statistics, and groups all brain regions showing synchronized source signals into independent components (ICs), which represent temporally coherent functional networks (FNs) (McKeown and Sejnowski, 1998; McKeown et al., 1998; Calhoun et al., 2002, 2009). McKeown et al. (1998) predicted that sICA would be more sensitive in detecting task-related changes in fMRI signal than the traditional general linear model (GLM) based analysis, because sICA uses a data-driven approach, and can reduce noise in the final solution by separating artifacts from real fMRI signal.

SICA has become widely used for fMRI analysis since its original application to fMRI 15 year ago (Calhoun and Adali, 2012). Most studies use sICA as a technique for extracting FNs from fMRI data, while some studies use sICA to separate and remove artifacts from real fMRI signal for improving the sensitivity of subsequent GLM based analysis (e.g., Aron and Poldrack, 2006). Several studies compared sICA and GLM based analyses and reported that sICA revealed more brain regions showing task-related activation relative to GLM based analysis, supporting the prediction of McKeown et al. (Calhoun et al., 2001; Malinen et al., 2007; Tie et al., 2008; Kim et al., 2011). Relevant to this issue, an interesting finding from sICA is overlap of two or more FNs of different timecourses and different task-related modulations. This finding has been interpreted as evidence of multiple concurrent central processes associated with common brain regions (Calhoun et al., 2008; Kim et al., 2009a,b; Menz et al., 2009; van Wageningen et al., 2009; Wu et al., 2009; St Jacques et al., 2011; Domagalik et al., 2012; Zhang and Li, 2012). Two recent studies, one from us (Xu et al., 2013) and the other from Beldzik et al. (2013) systematically analyzed FN overlap, and the task-related modulation of the timecourses of overlapping FNs. These analyses further highlighted the presence of brain functional activity hidden from the traditional GLM based analysis.

In Xu et al. (2013), sICA was used to extract FNs from fMRI data related to a visual target-identifying task. Most (~78%) functional brain regions showed overlap of two or more FNs. Several brain regions including the dorsal anterior cingulate (dACC), insula and adjacent ventrolateral prefrontal cortex (PFC), lateral temporal and parietal cortex, and precuneus/posterior cingulate (PCC) showed overlap of seven or more FNs. Each of overlapping FNs showed unique task-related modulations of timecourses, and some of them were opposite (i.e., increases vs. decreases) to each other. The prediction that such opposite modulations within the same voxels might be hidden from a GLM based analysis due to cancellation was tested by analyzing the same fMRI data using a GLM based analysis. The brain regions showing task-related activation and deactivation as revealed by the GLM based analysis are 11.9 and 26.2%, respectively, of brain regions showing corresponding changes as revealed by sICA, therefore supporting the prediction.

In Beldzik et al. (2013), the authors used both sICA and a GLM-based analysis to assess an fMRI dataset related to an anti-saccadic task. They developed a tool called Contributive Sources Analysis (CSA) for estimating the amplitude of fMRI signal changes in each FN. They first used the GLM-based analysis to define clusters showing significant task-related activity as regions of interests (ROIs) and then used CSA to extract measures of task-related changes in fMRI signal within these ROIs from all FNs overlapping with these ROIs. They confirmed their prediction that the sum of these measures from all FNs equals to the measure of task-related changes of fMRI signal within the ROIs as assessed by the GLM-based analysis. They further demonstrated that task-related opposite modulations of overlapping FNs contributed to the negative findings of the GLM based analysis at some brain regions. Therefore, findings from both studies are complementary, and indicate that multiple neural circuits, each with unique timecourse and task-related modulation, can occur concurrently within the same voxels/regions, and that this feature of brain functional organization may not be detected by GLM based analysis. This observation is consistent with several lines of evidence in the literature.

First, anatomical studies indicate that each voxel may contain multiple signal sources of different timecourses. For example, the human cortex has about 25–30,000 neurons/mm^3^ (Roth and Dicke, 2012), and each voxel may contain more than hundred thousands of excitatory and inhibitory neurons (Druga, 2009). They form multiple overlapping microcircuits. For example, each barrel column of the rat somatosensory cortex is about 300–500 μm in diameter and contains about 10 microcircuits (Brecht, 2007; Lubke and Feldmeyer, 2007). It receives two thalamic inputs of different response latencies, and generates five to seven outputs of various dynamics to different targets (Brecht, 2007). Therefore, it is reasonable to expect different microcircuits show different timecourses during processing of sensorimotor inputs. We acknowledge that whether and how the different timecourses of different microcircuits lead to different hemodynamic responses is not clear and should be investigated in future studies.

Second, electrophysiological studies indicate that neurons of different timecourses intermixed with each other in the same brain regions. For example, during the delay period of a working memory task, neurons in the dorsolateral PFC of monkeys show several patterns of activity changes including sustained increases in activity, increased activity at the beginning and gradually reduced activity later, and gradually increased activity (Chafee and Goldman-Rakic, 1998; Takeda and Funahashi, 2007; Verduzco-Flores et al., 2009). Furthermore, other neurons show various patterns of reduced activity during the delay period. It has been suggested that neurons showing different patterns of activity changes are associated with different cognitive processes such as cue representation, memory maintenance, and response preparation (Chafee and Goldman-Rakic, 1998; Verduzco-Flores et al., 2009). Based on these findings, Fuster hypothesized that different FNs overlap with each other in the brain (Fuster, 2009).

Third, published fMRI studies using analyses other than sICA report FN overlap. Yan et al. used a connected iterative scan (CIS) approach, which assesses FN overlap by detecting the involvement of the same functional clusters in different FNs, to assess FN overlap from fMRI acquired at resting condition (Yan et al., 2011). They reported overlap of the DMN with the so-called task-positive network at the PCC and lateral parietal cortices, consistent with the finding of overlapping FNs showing opposite modulation by sICA (Beldzik et al., 2013; Xu et al., 2013). A recent study applied temporal ICA to fMRI data and found that the DMN consists of multiple overlapping sub-networks (Smith et al., 2012). Several studies used seed-based approach to assess FNs from fMRI data and reported FN overlaps (Fox et al., 2005; Buckner et al., 2009; Tomasi and Volkow, 2011). The FN overlap reported in these studies may reflect the phenomenon of the timecourse of fMRI signal mixture in one region significantly correlating with the timecourses of two or more other regions, while the timecourses of the other regions do not significantly correlate with each other. However, the overlapping sICA components reflect different source signals from one brain region correlating with different source signals from two or more other brain regions.

The knowledge of FN overlap and their task-related modulation has important theoretical implications in understanding brain functional organization, and can help reconcile inconsistent data existing in the literature. For example, fMRI studies often show task-induced deactivation in the medial PFC and precuneus/PCC during tasks demanding working memory or cognitive control. It has been hypothesized that these brain regions are the core of DMN and associated with intrinsically generated, task-independent mental activity, e.g., task-unrelated thoughts (Fox et al., 2005; Raichle and Snyder, 2007). However, other findings indicate that these brain regions are also related to working memory. It has been reported that lesions of the medial PFC impair performance on working memory tasks (Tsuchida and Fellows, 2009; Barbey et al., 2011; Szatkowska et al., 2011). Furthermore, performance on working memory task correlates with the strength of functional connectivity within the DMN, especially between the medial PFC and precuneus/PCC (Hampson et al., 2006). The findings from the two sICA studies (Beldzik et al., 2013; Xu et al., 2013) of overlap of FNs with opposite task-related modulation indicate that there is no sharp border between neural substrates associated with cognitive control vs. task-unrelated thoughts, and that these neural substrates intermix with each other at both lateral and medial PFCs and precuneus/PCC. However, the lateral PFC may have more neural substrates associated with cognitive control, and fewer neural substrates associated with task-unrelated thoughts, and therefore, shows a net increase in fMRI signal mixture during task performance. On the other hand, the medial PFC may have more neural substrates associated with task-unrelated thoughts and fewer neural substrates associated with working memory, and therefore, shows a net decrease in fMRI signal mixture during task performance. This notion is supported by the electrophysiological evidence that neurons of one modality tend to concentrate in certain regions, but also distribute in other regions dominated by neurons of other modalities (Fuster, 2009).

In addition to the theoretical significance, the knowledge of FN overlap has important practical implications in how to assess brain functional organization. For example, seed-based approach is another popular technique for assessing FNs. This approach generates FNs by extracting the timecourse of fMRI signal from a selected voxel or ROI and then correlating this timecourse with the timecourses of other voxels or ROIs. Therefore, it does not consider different timecourses of different source signals from the same voxel or ROI, and thus may generate findings different from those by sICA, because some source signals from different ROIs may show significant correlations while the signal mixtures from these ROIs may show no significant correlations. Another example is that fMRI studies using GLM based analysis often report different changes in fMRI signal at some brain regions during task performance between patients and healthy controls. This difference in signal mixture could be due to changes in one or more source signals. The knowledge of changes in a specific source signal may provide further insight into etiology and neuropathology of the disease under investigation relative to the knowledge of changed signal mixture.

In summary, sICA can separate source signals from the same brain regions, and this specific capacity has not been fully exploited in most published fMRI studies. Two recent studies explicitly employed this capacity and revealed new insight into functional activity hidden from a GLM based analysis. These new insights have significant theoretical and practical implications in understanding brain functional organization. Thus, we recommend that investigators should use sICA or some other approach which can account for overlap cancelation to perform secondary analysis of published fMRI studies analyzed by GLM alone, and use both GLM and sICA in future fMRI studies for a more complete understanding brain functional organization.
